# The NIH must reduce disparities in funding to maximize its return on investments from taxpayers

**DOI:** 10.7554/eLife.34965

**Published:** 2018-03-23

**Authors:** Wayne P Wahls

**Affiliations:** Department of Biochemistry and Molecular BiologyUniversity of Arkansas for Medical SciencesLittle RockUnited States; eLifeUnited Kingdom

**Keywords:** science policy, federal funding, implicit bias, social prestige mechanisms, Matthew effect, Point of View, Human

## Abstract

New data from the NIH reveal that the scientific return on its sponsored research reaches a maximum at around $400,000 of annual support per principal investigator. We discuss the implications of this 'sweet spot' for funding policy, and propose that the NIH should limit both the minimum and maximum amount of funding per researcher.

The National Institutes of Health (NIH) is the federal steward of biomedical research in the United States. The NIH must ensure that scientists at-large are allowed to compete on equal footing for grant support, and it is obligated to allocate research dollars in a way that maximizes the returns on taxpayers’ investments. Two recent studies from within the NIH (Basson et al., 2016; Lauer et al., 2017) reveal with precision the perils of not doing so—and provide equally precise guidance for evidence-based changes in funding policy.

## Systemic disparities in funding

Principal investigators at-large do not have equal access to federal grant funding for scientific research and this chronic problem has long been recognized by federal funding agencies (National Academies, 2013). There are disparities in grant application success rates or award sizes or both for investigators grouped by race (Ginther et al., 2011), gender (Pohlhaus et al., 2011; Magua et al., 2017), age (Levitt and Levitt, 2017), institution (Murray et al., 2016) and state (Wahls, 2016a). To the extent tested these disparities persist even after controlling for other factors, suggesting that implicit (subconscious) biases and social prestige mechanisms (e.g., the Matthew effect) can affect allocations of funding. However, we do not need to define the various potential sources of bias, or even accept that there might be any bias at all in funding decisions, to understand that the unbalanced allocations of funding are detrimental to the biomedical research enterprise in the US.

**Figure 1.**
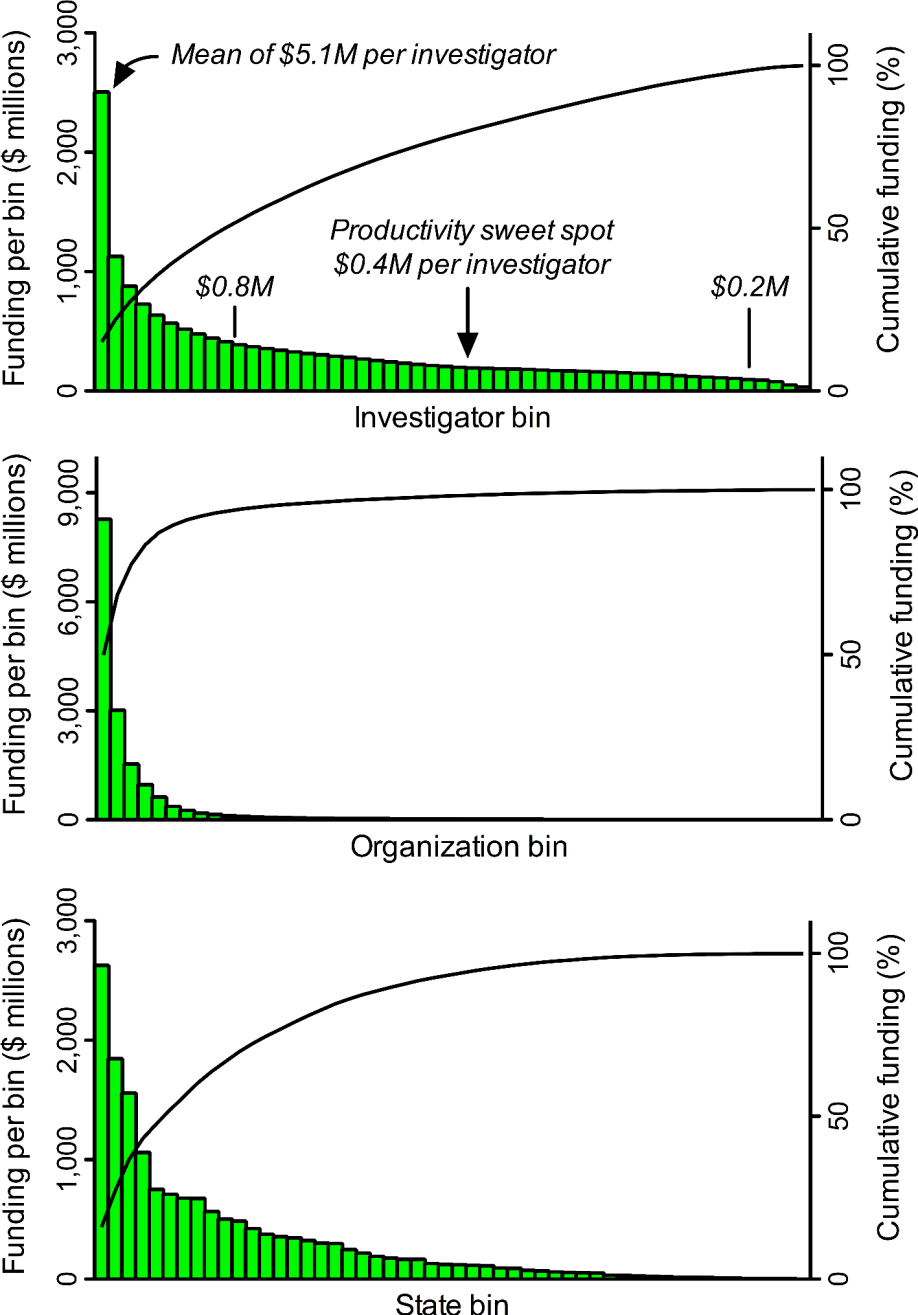
Heavily skewed distributions of NIH grant funding favor a minority and disfavor the majority. A search of the NIH RePORTER database identified 25,674 investigators who received research project grant funding in FY2015. These individuals were ranked in descending order by the amount of funding they received, and then grouped into 52 bins, each of which contained 493 investigators (the remaining, lowest-funded 38 investigators were not binned). The same process was applied for amounts of funding to 2,038 organizations (39 per bin) and to 52 states, including Washington DC and Puerto Rico (1 per bin). Pareto plots display amounts of funding (histograms, left Y axis) to each bin. For example, the first bin of investigators got more than twice as many dollars as the second bin. Cumulative curves (right Y axis) display fraction of total funding to a given bin and all higher-funded bins (i.e., those to its left). Inset text (italics) in the top panel show the mean amount of funding (in $ millions, M) per investigator for select bins. The amount of funding per investigator that yields maximum productivity (the 'sweet spot' from Figure 2) is almost exactly the median amount of funding per investigator. The proposed lower and upper limits for support per awardee ($0.2M and $0.8M) would free up enough money to support about 10,500 additional investigators (21 additional bins) with mean funding at the productivity sweet spot.

Differences in grant application success rates and award sizes (whose impacts on allocations of funding are multiplicative) contribute to heavily skewed distributions of funding that favor a small minority of scientists and disfavor the vast majority (Figure 1). Just 1% of NIH-funded investigators get 11% of research project grant dollars and 40% of the money goes to 10% of funded investigators (Collins, 2017a; amounts of funding include administrative supplements, if any). The distributions are even more heavily skewed at the level of institutions and states. While the NIH gives half of all research project grant dollars to about 19% of funded investigators, half the money goes to just 2% of funded organizations and 10% of states (Figure 1). These values underrepresent the true magnitude of disparity because many meritorious scientists who apply for support go unfunded.

Such “funding inequality has been rising since 1985, with a small segment of investigators and institutes getting an increasing proportion of funds, and investigators who start in the top funding ranks tend to stay there (which results in stasis, or lack of mobility)” (Katz and Matter, 2017). Elsewhere in the ranks, increasing hyper-competition for the limited resources places the majority of awardees at risk of laboratory closure when they lose their sole NIH grant (Peifer, 2017a). The hyper-competition also serves as an effective barrier for the recruitment of young investigators into the biomedical workforce, causing many highly talented trainees and early career scientists to redirect their career paths away from working on the underlying biology, diagnosis and treatment of human diseases (Carr, 2013). Consequently, thought-leaders and organizations such as the Federation of American Societies for Experimental Biology have advocated for a more equitable distribution of funding to help sustain the biomedical research enterprise (Alberts et al., 2014; Lorsch, 2015; FASEB, 2015). There are compelling reasons for such changes in funding policy.

## Double-edged sword of disparity

At population scale, the underfunding and non-funding of some groups of scientists (the majority) compromises their ability to contribute effectively to the missions of the NIH. It has been posited that a more balanced distribution of resources among investigators—geared towards harnessing the greatest possible number of perspectives, creative ideas and experimental approaches—would strengthen the diversity of the research ecosystem, increase the likelihood of scientific breakthroughs, and lead to a greater return on taxpayers’ investments (Lorsch, 2015). However, we must consider the alternative hypothesis. Is it possible that the negative impacts of underfunding the majority of investigators are offset by positive impacts of overfunding the minority?

In his 1985 commentary in *Cell*, Bruce Alberts pointed out that individual investigators each have a finite capacity to carry out grant-related duties and that their productivity falls when their amounts of funding exceed those capacity limits: consequently, highly funded laboratories are generally “less productive” and represent a “poor training environment” relative to more-modestly funded laboratories. Alberts advocated for capping the total amount of funding that each investigator can receive, explaining how this would reduce waste and would permit the funding of more investigators, thereby increasing the diversity and net productivity of the research enterprise (Alberts, 1985).

About three decades later, that prescient insight was validated by a series of empirical studies which showed that scientific output, as measured in multiple ways, does not scale uniformly with amounts of funding and that there are diminishing marginal (incremental) returns on investments in research (see, for example, Mongeon et al., 2016 and references therein). These diminishing marginal returns apply for the heavily skewed allocations of NIH funding among individual grants (Lauer, 2016a), investigators (Lauer et al., 2017), institutions (Wahls, 2016) and quartiles of states (Wahls, 2016a). Giving a disproportionately large share of grant funding to a minority of investigators, institutions and states is counterproductive—whether or not the imbalances are driven by bias.

## Maiden voyage of policy meets iceberg

Big ships turn slowly, but they can turn. In response to the plethora of data from groups within and outside of the NIH, in May 2017 the NIH director Francis Collins announced a new policy to cap funding per investigator (Collins, 2017a). This was a modest plan that would have capped the number of research awards (three R01 grant equivalents), not dollars, per investigator. The majority of very well-funded investigators would have been protected from the caps because only about one in five of the investigators with more than a million dollars of NIH research project grant funding per year has more than three such grants (Wahls, 2017). Nevertheless, according to Collins, the new policy would have freed up enough funds for about 1,600 new awards to help early and mid-career researchers who just miss the pay line for funding (Collins, 2017a). The “about 1,600 new awards” might seem like an impressive number, but it is actually a trivial increase given that the NIH supports almost 50,000 competitive grants to researchers.

One month later, in a stunning about-face that ignored the unanimity of conclusions from studies conducted within and outside of the NIH, the NIH cancelled the incipient policy on funding caps (Collins, 2017b). It was replaced by a plan (the Next Generation Researchers Initiative, or NGRI) that is predicted to, over five years, support up to 2,000 additional investigators (Collins, 2017b). The NGRI plan has no provisions to address the inefficient utilization of research dollars caused by the focused concentrations of funding at any of the levels described above.

## Sweet spot for funding; high cost of disparity

To what extent do the heavily skewed allocations of NIH research project grant funding affect the returns on taxpayers’ investments? Is there a specific amount of funding per investigator that yields optimal returns? Two recent studies conducted within the NIH provided important insight by measuring marginal returns (i.e., the incremental amount of productivity that is generated by each additional dollar of funding) for investigators with different amounts of NIH funding. One study used direct costs, the other used total costs. For those unfamiliar with the difference, each dollar of direct costs corresponds to about $1.50 of total costs (direct costs plus indirect costs). Although indirect cost rates vary between institutions, both direct costs and indirect costs go to support the research of a given project, so total costs provide the most appropriate parameter when it comes to measuring returns on taxpayers’ investments.

While there is no ideal, single way to measure scientific output and each metric has its caveats, the two NIH studies used broadly accepted measures. These are scientific publications and time-normalized citation impact factors per unit of funding. The latter metric encompasses the influence of the publications, as measured by how frequently other scientists cite the published work in their own articles, taking into account that article-level citation impact factors follow a log-normal distribution (see Hutchins et al., 2016 and references therein). Importantly, the various productivity metrics used in these studies (and others) support similar conclusions.

One study reported that scientific output for investigators funded by the NIH's National Institute of General Medical Sciences (NIGMS), based on the number of grant-weighted publications and citation rates, tapers off above $300,000 of annual direct costs and diminishes further thereafter, “with only small discontinuous increase above $500,000” (Basson et al., 2016). The impacts of these differences are staggering. Funding for a first R01 grant ($200,000 annual direct costs) to an investigator produces, on average, about five publications during the funding period, whereas the same amount of funding for a third R01 grant yields only about one additional publication (Lorsch, 2015). At least at population scale, about 80% of the dollars allocated for each third grant to an investigator are not being used productively, relative to what could be realized by giving that grant to an unfunded investigator (of which there are many, given that about three quarters of applicants are denied funding each year; Rockey, 2014).

**Figure 2.**
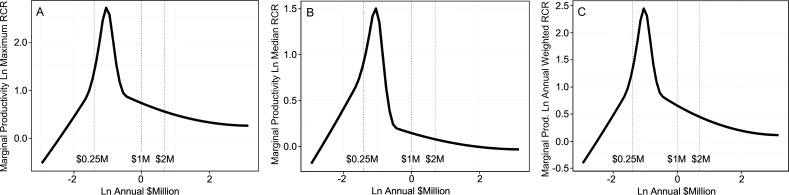
Productivity peaks at about $400,000 per investigator and declines with lower and higher amounts of funding. Each plot shows the marginal return (Y axis) as a function of annual NIH research project grant funding (total costs) per investigator (X axis); note that both axes are logarithmic, and that the range of the Y-axis varies from plot to plot. The marginal return for each amount of funding corresponds to the first derivative of the Cobb-Douglas production function, using relative citation ratios (RCRs) as the measure of production (Mongeon et al., 2016; Lauer et al., 2017). The RCR is a measure of article influence, developed by the NIH, that normalizes the number of citations received by a publication for the field of study and the time of publication (Hutchins et al., 2016). The three plots show the marginal return based on the maximum RCR (**A**), median RCR (**B**) and annual weighted RCR (**C**). The vertical dashed lines correspond to funding values of $250,000, $1 million and $2 million per investigator. Reproduced with permission and minor modifications (increased font size and line weights, repositioned panels and labels) from (Lauer et al., 2017) under a CC-BY 4.0 international license.

The second study extended the analyses to include all NIH-funded investigators and came to essentially identical conclusions (Lauer et al., 2017). Optimal rates of output, based on time-normalized, field-normalized citation impact factors, occur at about $400,000 of total costs per investigator (the sweet spot for funding; Figure 2). That sweet spot coincides almost exactly with median funding per investigator (Figure 1). However, there are diminishing marginal returns for amounts of funding above and below that sweet spot. For example, the incremental returns for each dollar of funding at $800,000 (twice the optimal level) and at $200,000 (half the optimal level) are about 25% to 40% of the returns at the sweet spot (Figure 2). Thus the majority of the dollars allocated outside of this range are not being used effectively, relative to what could be realized by funding investigators at the sweet spot. Notably, the diminishing marginal returns persist even when award data are parsed by NIH institute, for “elite” investigators, and by human versus non-human model systems (Lauer et al., 2017).

## NIH should limit minimum and maximum funding per investigator

The findings from both of the recent NIH studies, and many others, further support the calls from thousands of concerned individuals (including an online petition: Peifer, 2017c) for the NIH to reinstate or otherwise impose an upper limit on funding per investigator. Caps based on the number of grants per investigator would be ineffective at addressing the unbalanced allocations of funding because, under a three-grant cap (Collins, 2017a), about 80% of investigators with more than a million dollars of support per year would be protected from any reductions in funding (Wahls, 2017). There would be little impact on the heavily skewed allocations of funding and, correspondingly, upon the diminishing marginal returns. Even a more stringent, two-grant cap would have no effect on amounts of support to about half of NIH’s millionaires. Therefore, the caps should be based on dollars and should be applied uniformly to all awardees with very few or no exceptions (Alberts, 1985).

Dollar-based funding caps would be effective. For example, a cap of one million dollars (total costs) of annual NIH research project grant funding per investigator—which is an extremely generous amount of support that is far beyond the point at which diminishing marginal returns kick in (see Figure 2)—would reduce inefficiencies at the top end of the funding distribution and would free up enough money to support about 10,000 additional investigators (Wahls, 2017). This would be far more effective at expanding the investigator pool and at rescuing early to mid-career investigators than the new NGRI program, which will not address differences in productivity per dollar of funding and is expected to fund only about 2,000 additional awards over the next five years (without any clear indication of award sizes or where the dollars will come from; Collins, 2017b).

Notably, investigators with amounts of NIH funding below the sweet spot also have sub-optimal productivity (Figure 2; Lauer et al., 2017), presumably because they lack the critical mass (e.g., number of grant-supported personnel) to sustain high productivity. The double-edged sword of disparities in allocations of funding is sharp on both edges, each of which cuts the efficiency with which precious research dollars are being expended.

I therefore call on the NIH to establish both a lower limit and an upper limit for the amount of NIH research project grant funding per awardee each year. I posit, based on available data (such as Figure 2) and as a point for discussion, that a lower limit of $200,000 and an upper limit of $800,000 total costs would be appropriate. Within this range, median funding per investigator would still be close to the productivity sweet spot of $400,000 (Figure 1). The flanking limits would increase returns on taxpayers’ investments by curbing inefficiencies at the low and high ends of the funding distribution, while still affording considerable flexibility in amounts of support to each investigator. The large (four-fold) range in allowed funding would accommodate the fact that some types of research are more expensive than others. Moreover, these specific limits would free up enough money to support more than 10,000 additional investigators, each funded at (or with mean funding at) the productivity sweet spot of $400,000. Here’s why:

Based on FY2015 values from NIH RePORTER, 5,038 research project grant recipients (which is 20% of the total number of grant recipients) each got more than $800,000. Together they received about $8.39 billion, so limiting each to $800,000 would free up about $4.36 billion. There were 2,302 investigators (9% of the total) with funding between $10,000 and $199,999. Together those awardees got $0.32 billion, so bringing each of them up to the $200,000 minimum would cost about $0.14 billion. Thus the minimum and maximum funding limits would free up about $4.22 billion, which is enough to award $400,000 to each of 10,542 additional investigators who do not have funding.

## Harnessing additional talent and its limitations

To what extent would supporting about 10,500 additional investigators expand the funded workforce? Is there an existing capacity to support such changes? Would the NIH still be funding only meritorious research projects? These and related questions can be answered by comparing calculated impacts of the proposed funding limits to available data.

When measured over five-year periods ending in 2003 and 2015, the number of NIH research project grant applicants rose from about 60,000 to slightly less than 90,000, but the number of awardees held steady at about 27,500 (Lauer, 2016b). Over the same time frame (2003 to 2015), the value of the NIH budget not only failed to keep pace with the expanding US population in general, and the expanding scientific workforce in particular, it actually lost 22% of its purchasing power due to budget cuts, sequestration and inflation (FASEB, 2017). For these reasons, grant application success rates and investigator funding rates have fallen fairly steadily over time.

Each year only about one quarter of applicants, including those who submit multiple proposals, get funded (Rockey, 2014). Similarly, less than one third of applicants secure any research project grant funding over a five-year period (up to 2015); about 60,000 applicants do not get any of their applications funded (Lauer, 2016b). Therefore, the unutilized capacity of the biomedical workforce (as measured by unfunded applicants) is large enough to sustain an additional 10,500 awardees, which would increase the pool of funded investigators by about 38%. Competition for funding would remain fierce, the additional awards would go only to meritorious investigators whose applications receive high priority scores from scientific peer review, and the majority of applicants would remain unfunded.

It thus seems clear that while dollar-based funding limits would be quite effective at harnessing additional talent, in isolation they would be insufficient to address the problem of too many investigators competing for too few research dollars. Additional steps, such as restoring the NIH budget to its inflation-adjusted 2003 levels or even higher (to account for population expansion), would be required to ensure that biomedical research in the US remains competitive on the international stage. Meanwhile, concerned scientists are discussing ideas and principles, and agency officials are exploring additional mechanisms, to support a robust research ecosystem in the face of finite resources (see, for example, Levitt and Levitt, 2017; Alberts et al., 2014; Lorsch, 2015; FASEB, 2015; Pickett et al., 2015; Blume-Kohout and Adhikari, 2016; Schaller et al., 2017; Heggeness et al., 2017; Plank-Bazinet et al., 2017). A good example is the Maximizing Investigators’ Research Award (MIRA) program, a NIGMS program to “fund people, not projects” (NIGMS, 2017). The MIRA program seeks to increase the efficiency of funding by providing investigators with greater stability and flexibility while distributing funding more widely among investigators. To participate in this program, MIRA awardees must agree to accept the MIRA grant as their sole source of NIGMS research funding. The MIRA program, coupled with clearly defined limits on dollars of support per investigator, could serve as a paradigm for all NIH research project grant funding.

Giving a disproportionately large share of grant funding to a minority of investigators, institutions and states is counterproductive—whether or not the imbalances are driven by bias.

## Use data, not power of affluence, to guide policy

Sustaining the competitiveness of biomedical research in the US, and the benefits it brings to US citizens, can only be maintained through adequate appropriations for the NIH budget. Congress began restoring the NIH budget in FY2016 (FASEB, 2017) and all of us should encourage them to keep doing so. We should also help to ensure that the investments are utilized as efficiently as possible. To do this we must be critical (in a positive, analytical sense) of extant funding mechanisms, incipient programs and proposed changes to funding policy, including those put forth in this article. I therefore propose an overarching, guiding principle: Policies aimed at sustaining the biomedical research enterprise and for maximizing the efficiency with which research dollars are expended will be most effective only if they address adequately the vast disparities in funding among investigators, institutions and states.

Understandably, individuals who benefit directly or indirectly from unbalanced, heavily skewed allocations of funding (Figure 1) will campaign to preserve the status quo. Moreover, as a general rule in societies, affluence confers political power. The NIH’s rapid cancellation of its incipient, evidence-based, modest plan to cap funding per investigator—seemingly in response to “a concerted effort by a few very well-funded and powerful scientists threatened by this new approach, combined with a failure of the rest of us to vocally support the underlying idea…” (Peifer, 2017b)—is an excellent case in point. The “rest of us” who assumed that the policy would be implemented had no compelling reason to voice our opinions at the time of its announcement, speaking up en masse (see, for example, Peifer, 2017c) only once the incipient policy was, unexpectedly, cancelled. Importantly, there is no scientific basis for the NIH to capitulate to the wishes of the affluent minority. A plethora of data from within and outside of the NIH document unambiguously the perils of giving the majority of funding to a minority of investigators—and those data provide benchmarks for remediation through changes in funding policy.

## Empirical imperatives

Strong disparities in allocations of federal funding for scientific research, such as those shown in Figure 1, are deleterious because they degrade the diversity and productivity of the research enterprise. While the etiology of this problem might be complex, there is a straightforward, effective mechanism that can provide substantial remediation for many of its consequences:

- To address the inefficiencies caused by heavily skewed allocations of funding;
- To distribute grants and grant dollars more equitably among investigators, institutions and states;
- To rescue talented early and mid-career researchers who just miss out on funding;
- To provide a more reliable stream of support for the approximately 70% of investigators whose laboratories subsist on a single grant;
- To harness the creative ideas of additional, meritorious investigators at all levels who are victims of abysmal funding rates (untapped talent and capacity);
- And to maximize the returns on taxpayers’ investments—

The NIH must cap the number of research project grant dollars that each investigator can receive and it should also consider establishing a minimum amount of support per awardee.
