# Supplementary material for: The NIH must reduce disparities in funding to maximize its return on investments from taxpayers
Source: eLife. 2018 Mar 23;7:e34965. doi: 10.7554/eLife.34965 (PMC5882298; doi:10.7554/eLife.34965)
Supplement: Peer review history

# Decision letter

**DOI:** 10.7554/eLife.34965.005

**Affiliations:** eLifeUnited Kingdom

In the interests of transparency, eLife includes the editorial decision letter and accompanying author responses. A lightly edited version of the letter sent to the authors after peer review is shown, indicating the most substantive concerns; minor comments are not usually included.

Thank you for submitting your article "Point of View: To maximize returns on taxpayers' investments the NIH must reduce disparities in funding" to *eLife* for consideration as a Feature Article. Your article has been reviewed by three peer reviewers, and the evaluation has been overseen by the *eLife* Features Editor. The following individuals involved in review of your submission have agreed to reveal their identity: Mark Peifer (Reviewer #1).

Overall the reviewers were positive about the article, but they had a number of concerns (see below). We therefore invite you to prepare a revised submission that addresses these concerns.

Summary:

This is a provocative opinion piece that argues that, based on data on direct costs from NIGMS and total costs from NIH, there is a "sweet spot" of funding: $400,000 total costs. This sweet spot defines an optimal return on taxpayers' investments. Based on this sweet spot, the author argues that the NIH should install not only a cap on NIH funding ($800,000 total costs per investigator) but a minimum as well ($200,000 per investigator). Further, the author points out that the installation of this maximum and minimum would free up enough funds to allow the funding of about 10,000 more investigators, substantially diversifying the investigator pool that participates in NIH funded research.

This is an important editorial that should be published to generate further conversation. However, there are a number of issues that the author should address.

Essential revisions:

1) It's unclear to this reviewer why the author focuses on total costs versus direct costs. There is a dramatic range in indirect costs among institutions and having a cap defined by total costs essentially introduces a new tiered funding model that is defined by where an investigator does their research. Focusing on direct costs seems a more equitable solution to increasing the diversity of the investigator pool. Please revise the manuscript to either focus on direct costs or explain why you focus on total rather than direct costs.

2) It's not clear what the minimum funding level is meant to accomplish. I identified 2019 R01s that were funded at less than $200,000 in 2015 but half of these (1154) were funded administrative supplements, which are explicitly meant to provide additional funding to meet increased costs. With only ~800 R01s funded at levels below $200,000 in 2015, I don't see a compelling reason why this population of investigators requires intervention.

3) One of my concerns is that the whole argument takes for granted the number of papers authored by an investigator are a good indicator of this individual contribution to this "return on taxpayers' investments". In my view it should be stressed in the paper that (perhaps especially in today's publish or perish environment) that the number of publications is not a great measure of the overall outcome of research.

4) We already know that there are diminishing returns to increased research funding in terms of research output and impact. We also know that medial research is not a simple "dollars in – papers out" machine. It is way more complex than that. Accordingly, there is a body of literature that addressed the multiple challenges of the research system and proposed solutions, among which the capping of research funding (See for example: https://doi.org/10.1073/pnas.1509901112). Please clearly state the new insights provided by this manuscript.

5) I think the argument would be strengthened if numbers of investigators seeking grants would be included. Funding an additional 2,000 or 10,000 investigators sounds good, but how closer does that bring us to funding everybody? How much would it cost to simply give $200,000/year to every investigator? Is it realistic/feasible? Perhaps the number reported here could be useful: https://nexus.od.nih.gov/all/2016/05/31/how-many-researchers/

6) Re tone and choice of words: I think it is important to allow his passion for the subject to come through, but repeatedly using the word "waste" to describe the impact of the current funding scheme is both inaccurate and potentially damaging to our enterprise. I would strongly suggest that the author temper his tone in this regard. The same point can be made by talking about maximizing productivity, without inadvertently providing ammunition to some who might want to reduce our nation's investment in basic science. These can be found throughout the text.

7) I also would suggest the author include a paragraph describing the current efforts of the NGRI Working Group, mentioning the original policy and perhaps critiquing it.

8) Figure 1 needs to be explained more clearly. In the caption, please first explain Figure 1A in detail (saying that the XXXXX investigators funded by the NIH were ranked according to amount of funding, and then grouped in 50 bins (each containing XXXXX/50 investigators) etc. Figure 1B and Figure 1C can then be explained more succinctly.

9) Please consider showing the three curves in different colours on a single set of axes. Also, please give the actual amount of funding in the labels for the x-axis, and explain in the figure caption that this axis is logarithmic. Also, please explain the three y-axes more fully in the caption.

# Reply: Author response

**DOI:** 10.7554/eLife.34965.006

Essential revisions:

1) It's unclear to this reviewer why the author focuses on total costs versus direct costs. There is a dramatic range in indirect costs among institutions and having a cap defined by total costs essentially introduces a new tiered funding model that is defined by where an investigator does their research. Focusing on direct costs seems a more equitable solution to increasing the diversity of the investigator pool. Please revise the manuscript to either focus on direct costs or explain why you focus on total rather than direct costs.

It is necessary to refer to both direct costs and total costs (direct plus indirect) because these are the metrics used in the two key papers that are discussed. I agree to explain why the other discussions focus on total costs and have done so.

2) It's not clear what the minimum funding level is meant to accomplish. I identified 2019 R01s that were funded at less than $200,000 in 2015 but half of these (1154) were funded administrative supplements, which are explicitly meant to provide additional funding to meet increased costs. With only ~800 R01s funded at levels below $200,000 in 2015, I don't see a compelling reason why this population of investigators requires intervention.

The cited and discussed data are for all research project grants, not just R01s. The relevant values are the amount of funding per investigator, not the amount of funding per grant. Funding per investigator includes their sum for award(s) and administrative supplement(s), if any. I clarified this in the text.

Yes, the number of investigators with less than $200,000 per year is smaller than the number investigators with greater than $800,000 per year. But if we are to address inefficiencies at the top end of the funding distribution, it seems necessary and appropriate to address inefficiencies at the bottom end, too. For clarity, I describe both the numbers of investigators and the amounts of funding affected by limits at the top end (5,038 PIs, $4.36 billion) and at the bottom end (2,302 PIs, $0.14 billion). While some individuals might consider a mean annual increase of about $60,000 per investigator to be trivial, it is actually a very large percentage increase for investigators at the bottom end of the funding distribution. This type of intervention, which involves many investigators, is fully warranted by the differences in productivity.

3) One of my concerns is that the whole argument takes for granted the number of papers authored by an investigator are a good indicator of this individual contribution to this "return on taxpayers' investments". In my view it should be stressed in the paper that (perhaps especially in today's publish or perish environment) that the number of publications is not a great measure of the overall outcome of research.

Iagree that there is no ideal, single way to measure scientific output and that each metric has its caveats. I now describe this explicitly, as suggested, along with a statement that the two NIH studies used broadly accepted measures of productivity. Please note that the “whole argument” is based on more than just publication rates. I also describe how citation impact factors (used by both NIH studies) provide a measure of article influence that goes beyond simply counting publications. Importantly, the various productivity metrics used in these studies (and others) support similar conclusions. I now state this in the manuscript.

4) We already know that there are diminishing returns to increased research funding in terms of research output and impact. We also know that medial research is not a simple "dollars in – papers out" machine. It is way more complex than that. Accordingly, there is a body of literature that addressed the multiple challenges of the research system and proposed solutions, among which the capping of research funding (See for example: https://doi.org/10.1073/pnas.1509901112). Please clearly state the new insights provided by this manuscript.

I revised the text to state more clearly the new insights, as requested. For additional insight, I now describe in quantitative terms why the proposed funding limits alone would be inadequate to address the “multiple challenges” of the research system. The added text includes consideration of other “proposed solutions”, such as those in the PNAS article whose link was provided. Lastly, to further emphasize the new insight, “I propose an overarching, guiding principle” that is germane to all policies aimed at sustaining the biomedical research enterprise or for maximizing the efficiency with which finite research dollars are expended.

5) I think the argument would be strengthened if numbers of investigators seeking grants would be included. Funding an additional 2,000 or 10,000 investigators sounds good, but how closer does that bring us to funding everybody? How much would it cost to simply give $200,000/year to every investigator? Is it realistic/feasible? Perhaps the number reported here could be useful: https://nexus.od.nih.gov/all/2016/05/31/how-many-researchers/

This is an excellent suggestion. I now describe numbers of applicants and investigator funding rates. I report that 10,500 additional awards with mean funding of $400,000 would increase the number of funded investigators by about 38%. However, the majority of investigators would remain unfunded. I discuss relevance to existing, unutilized capacity of the workforce and to additional proposed remedies (see response to point 4).

If the NIH gave exactly (or on average) $200,000 to each investigator, there would still be unfunded applicants. I did not include this calculation in the article because: (A) that amount of funding is unrealistically low relative to the productivity sweet spot; and (B) amounts of funding per investigator must cover a broad range to accommodate the fact that some types of research cost more than others.

6) Re tone and choice of words: I think it is important to allow his passion for the subject to come through, but repeatedly using the word "waste" to describe the impact of the current funding scheme is both inaccurate and potentially damaging to our enterprise. I would strongly suggest that the author temper his tone in this regard. The same point can be made by talking about maximizing productivity, without inadvertently providing ammunition to some who might want to reduce our nation's investment in basic science. These can be found throughout the text.

I thank the reviewer(s) for this valuable insight. I replaced each of the direct statements with more innocuous terms.

7) I also would suggest the author include a paragraph describing the current efforts of the NGRI Working Group, mentioning the original policy and perhaps critiquing it.

I now described aspects of the NGRI program and the MIRA program that are directly relevant to the proposed funding limits. Space limitations and scope of the article (which is already well beyond the recommended length and number of references) preclude critiquing such programs in detail.

8) Figure 1 needs to be explained more clearly. In the caption, please first explain Figure 1A in detail (saying that the XXXXX investigators funded by the NIH were ranked according to amount of funding, and then grouped in 50 bins (each containing XXXXX/50 investigators) etc. Figure 1B and Figure 1C can then be explained more succinctly.

As suggested, I revised the legend of Figure 1 to enhance clarity.

9) Please consider showing the three curves in different colours on a single set of axes. Also, please give the actual amount of funding in the labels for the x-axis, and explain in the figure caption that this axis is logarithmic. Also, please explain the three y-axes more fully in the caption.

I revised the legend of Figure 2 to better explain the axes’ labels and the use of logarithmic values. (A cited basis for using a log scale was also added to the text.) This figure is reproduced with permission from another study, so I think it would be best to display the figure in unaltered format (i.e., as produced by the authors of that study).
